# Half-hourly ground surface temperature dataset from 24 sites in Wudaoliang, Qinghai–Tibet Plateau, 2019–2023

**DOI:** 10.1038/s41597-026-07925-6

**Published:** 2026-07-17

**Authors:** Defu Zou, Tianjie Zhao, Lin Zhao, Zanpin Xing, Xiaofan Zhu, Guojie Hu, Tonghua Wu, Xiaodong Wu, Yifan Wu, Defeng Feng, Jingyao Zheng, Shaojie Du, Zhen Wang, Ning Li, Chi Wang, Jimin Yao, Yao Xiao, Erji Du, Guangyue Liu

**Affiliations:** 1 Cryosphere Research Station on the Qinghai–Tibet Plateau, State Key Laboratory of Cryospheric Science and Frozen Soil Engineering, Northwest Institute of Eco–Environment and Resources, Chinese Academy of Sciences, Lanzhou, 730000 China; 2 State Key Laboratory of Remote Sensing and Digital Earth, Aerospace Information Research Institute, Chinese Academy of Sciences, Beijing, 100101 China; 3 School of Geographical Sciences, Nanjing University of Information Science and Technology, Nanjing, 210044 China; 4 Center for Pan–Third Pole Environment, Lanzhou University, Lanzhou, 730000 China; 5 China Satellite Communications Company Limited, Beijing, 100190 China; 6 National Geomatics Center of China, Beijing, 100830 China; 7 College of Surveying and Geoinformatics, Tongji University, Shanghai, 200092 China

## Abstract

Ground surface temperature (GST) is an essential boundary condition for permafrost modeling and satellite-product evaluation, yet high-frequency, multi-year GST observations on the Qinghai–Tibet Plateau (QTP) remain sparse, particularly within the interior continuous permafrost zone. Here we present a publicly available dataset of half-hourly GST measured at 24 sites in the Wudaoliang region of the central QTP. The network spans three dominant alpine land-cover types (alpine desert, alpine steppe, and alpine meadow) and elevations of 4,543–4,741 m a.s.l. HOBO temperature loggers were installed at 3–5 cm depth and recorded GST at 30-minute intervals from September 2019 to September 2023. The time series were quality controlled using physical-range screening and change-point checks, and disturbance-affected segments associated with frost-jacking were removed, yielding 1,399,008 valid records. The release includes quality-controlled GST time series, site-level metadata and a traceable exclusion log. This dataset supports reuse for the site- and grid-scale evaluation and calibration of satellite land surface temperature and freeze–thaw products, as well as studies of near-surface ground thermal dynamics in high-elevation permafrost regions.

## Background & Summary

Ground surface temperature (GST) is a fundamental boundary condition that governs land–atmosphere energy exchange and is closely linked to the thermal state of permafrost^1,2^. In widely used permafrost models—including the Stefan and Kudryavtsev models, the Temperature at the Top of Permafrost (TTOP) scheme, and the Geophysical Institute Permafrost Laboratory (GIPL) model—GST exerts first-order control on active layer dynamics and long-term permafrost evolution^3–6^. Continuous GST records are also essential for validating and calibrating satellite-derived land surface temperature (LST) products and atmospheric reanalyses^7–10^. These broad applications underscore the growing need for long-term, high-frequency GST datasets that support both process-based simulations and multi-scale evaluations.

Over recent decades, global initiatives such as the Circumpolar Active Layer Monitoring (CALM) network^11^, the Global Terrestrial Network for Permafrost (GTN-P)^12^, and the Permafrost and Climate in Europe (PACE)^13^ project have considerably advanced permafrost monitoring. However, these networks remain strongly biased toward Arctic and sub-Arctic regions. On the Qinghai–Tibet Plateau (QTP), the world’s largest high-altitude permafrost region, GST observations are comparatively sparse. Existing data mainly come from national meteorological stations at accessible lower elevations, leaving much of the continuous permafrost zone critically underrepresented^14,15^. Early GST measurements based on manual observations over snow or bare ground introduced systematic biases^16,17^. Although automated sensors, introduced in the early 2000s, improved accuracy, the fundamental limitation of sparse coverage persists^18^.

Recent efforts in the eastern QTP have begun to narrow this gap. Luo *et al*. (2019) established a 22-site network in the Bayan Har Mountains and reported a GST lapse rate of 1.1 °C per 100 m based on one year of hourly measurements^2^. More recently, Şerban *et al*. (2024) further extended observations in this broader mountain region by deploying 72 probes and recording GST every three hours over two consecutive years, providing valuable insights into how surface variability modulates near-surface ground temperature and permafrost occurrence^19^. Collectively, these studies have substantially improved the field-based understanding of GST variability in the eastern QTP mountain regions. In contrast, comparable field-based GST observations remain limited in the central QTP, where higher elevations, expansive plains, and continuous permafrost are dominant^20,21^. In such settings, dense, high-frequency observations across representative land-cover types are needed to quantify cross-surface contrasts and local-scale variability in ground thermal conditions.

Situated in the central QTP, the Wudaoliang (WDL) region comprises broad, high-elevation plains where the dominant land-surface types experience comparable synoptic forcing, providing a favorable setting for cross-surface comparisons of GST. WDL has long served as a permafrost research hub, with extensive observations of natural systems and engineering infrastructure^22–25^. However, existing observations have not yet systematically characterized GST contrasts among the dominant land-cover types or their relevance to permafrost thermal conditions in WDL. The region is underlain by continuous, often ice-rich permafrost; boreholes reveal substantial excess ice in the upper permafrost and additional ice-rich horizons at depths of several meters^26,27^. Recent studies have documented ongoing ground warming and permafrost degradation in this region^28–32^, with implications for the stability of critical linear infrastructure corridors crossing WDL^33–35^. This combination of regional significance, ongoing permafrost change, and the remaining GST observation gap motivates the release of open, quality-controlled GST observations to document the evolving ground thermal regime and support model- and satellite-based evaluations.

Leveraging this setting, a dense GST monitoring network was deployed in WDL and integrated with the Soil Moisture Network in Wudaoliang (SMN-WDL)^36^. The resulting dataset provides multi-year GST time series at 30-minute intervals from 24 sites spanning alpine desert, alpine steppe, and alpine meadow land-cover types within WDL. Alongside the quality-controlled GST series and site-level metadata, a traceable event log documents excluded periods and their causes, allowing users to interpret data gaps and reproduce the exclusion mask and the analyses reported in this Data Descriptor. Compared with prior regional datasets sampled every 1–3 h, the 30-minute resolution supports robust characterization of diurnal cycles, freeze–thaw transitions, and short-lived extremes while maintaining multi-year continuity. This Data Descriptor presents the network design, instrumentation, and quality-control workflow; documents the dataset structure, spatiotemporal coverage, and key statistics across land-cover types; and outlines recommended reuse for evaluating and calibrating satellite LST and freeze–thaw products and for permafrost and land–atmosphere thermal process studies under high-elevation conditions.

## Methods

### Study area

**Fig. 1:**
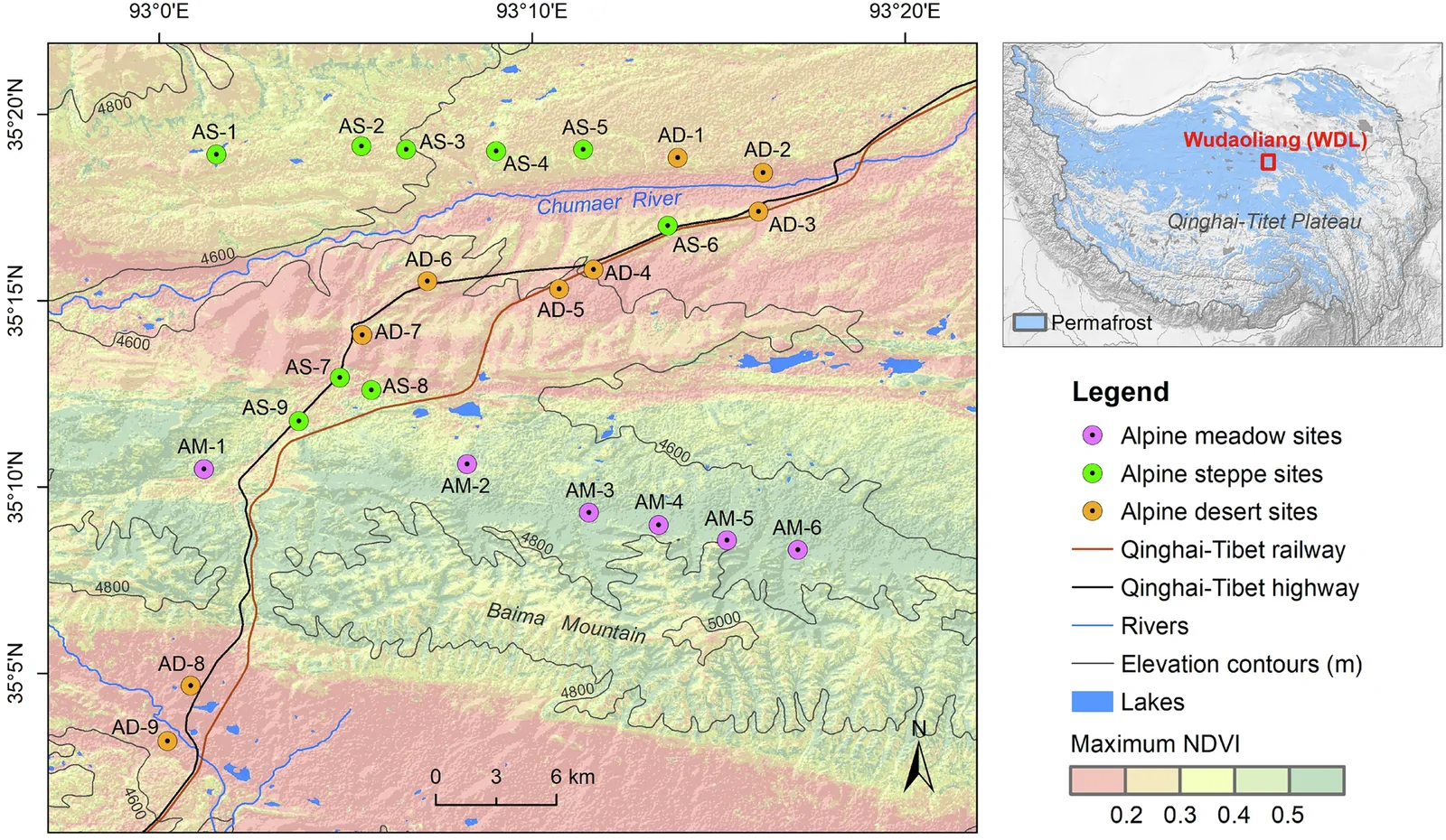
The study area and the locations of the 24 ground surface temperature (GST) sites in the Wudaoliang (WDL) region, central Qinghai–Tibet Plateau (QTP). The background shows a maximum-value NDVI composite (2019–2023) derived from Landsat 8 OLI surface reflectance. Elevation contours are shown at 200 m intervals. The inset shows the location of WDL within the continuous permafrost region of the QTP, based on Zou *et al*.^9^.

The Wudaoliang (WDL) region lies in the central QTP within the Qinghai‒Tibet Engineering Corridor (QTEC), spanning 93.0–93.3°E and 35.0–35.3°N (Fig. 1). The area has a typical continental plateau climate, with a mean annual air temperature of approximately −5.1 °C and mean annual precipitation of 299.8 mm, most of which occurs between May and September^37^. Elevations range from 4,527 to 5,118 m a.s.l., comprising broad alluvial plains and low-relief uplands in a transitional zone between semi-humid and semi-arid regimes.

Vegetation distribution in WDL is largely controlled by topography and soil moisture, resulting in distinct spatial patterns of the dominant land-cover types. Lower-elevation alluvial plains in the northern and southern parts of the region, along the Chumaer and Honglianghe rivers, are dominated by alpine desert (AD) and alpine steppe (AS) communities developed on coarse-textured, sandy soils. In contrast, the central and southern uplands, with higher elevations and steeper terrain, are dominated by alpine meadow (AM) on finer, clay-rich soils with a higher moisture content. This gradient in elevation, soil properties, and vegetation informed the stratified placement of the 24 GST sites across AD, AS, and AM (Fig. 1).

### Network design and layout

**Fig. 2:**
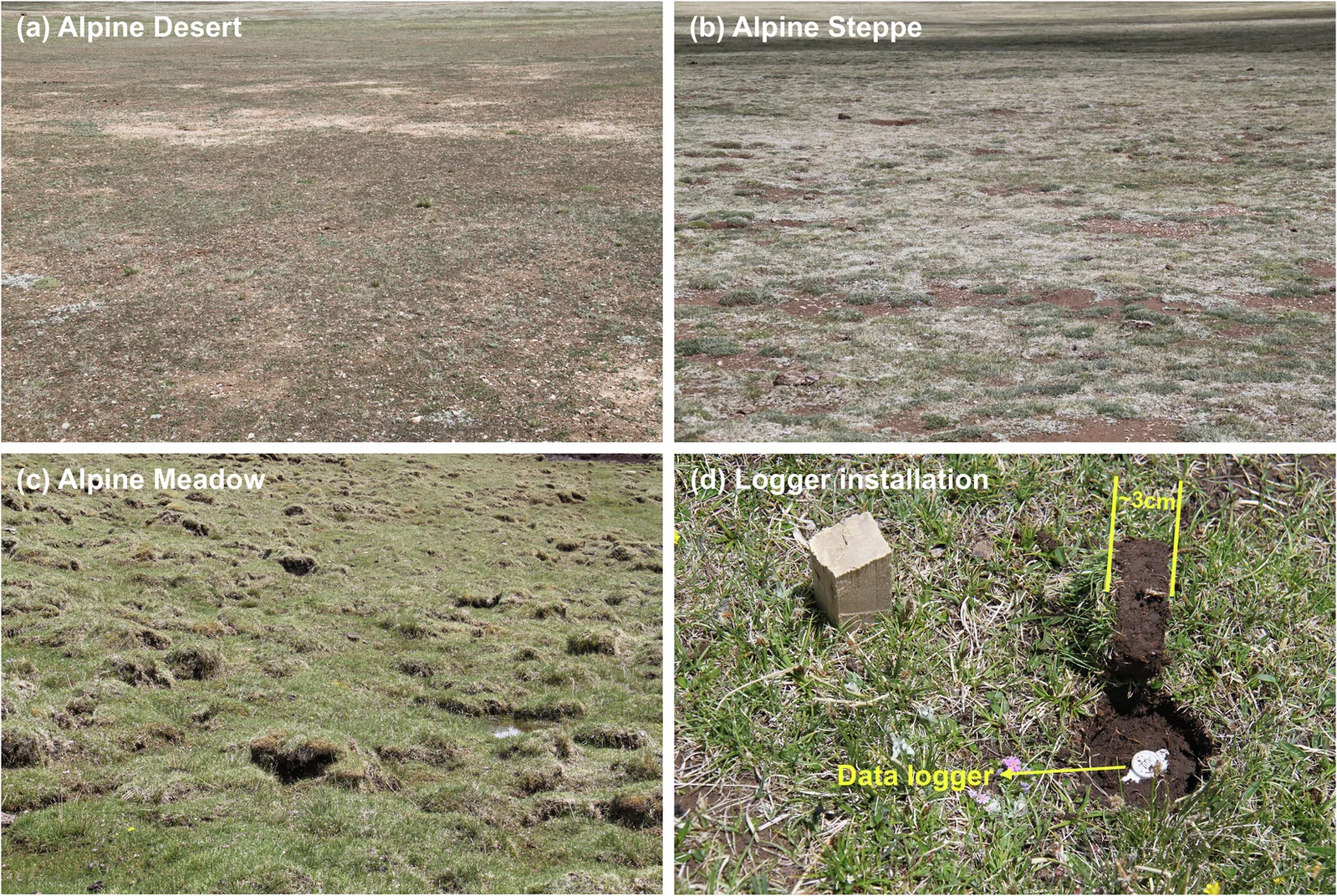
Representative site conditions and sensor installation in the Wudaoliang (WDL) region. Photographs show the (**a**) alpine desert (AD), (**b**) alpine steppe (AS), (**c**) alpine meadow (AM), and (**d**) installation of a HOBO MX2203 logger at a depth of 3–5 cm in a hand-augered soil pit.

During September–October 2019, a 24-site GST network was installed across WDL to augment the SMN-WDL, a 10-station network with vertically profiled soil hydrothermal measurements^36^. In September 2019, GST loggers were co-located at these 10 stations to add high-frequency near-surface temperature observations, and in October, the network was expanded with additional sites to densify spatial coverage across WDL. The network design targets the three dominant land-cover types—AD (9 sites), AS (9 sites), and AM (6 sites)—to enable controlled cross-surface comparisons. Land-cover type at each site was assigned based on field surveys, using local surface characteristics and dominant vegetation as the primary criteria. Representative species include *Ceratoides compacta* and *Saussurea arenaria* in AD, *Carex moorcroftii* and *Stipa purpurea* in AS, and *Kobresia pygmaea* and *Kobresia humilis* in AM^38^ (Fig. 2).

The network footprint spans approximately 36 × 36 km and overlaps approximately 16 grid cells of the 9-km Equal-Area Scalable Earth Grid 2.0 (EASE-Grid 2.0). The layout comprises one north–south transect aligned with the Qinghai–Tibet Highway and Railway, and two east–west transects following the Chumaer River and Baima Mountain. Site elevations range from 4,543 to 4,741 m a.s.l. AD and AS sites occur at similar elevations, with mean elevations of 4,613 and 4,623 m a.s.l. and elevation ranges of 149 and 119 m, respectively, whereas AM sites are higher on average, with a mean elevation of 4,694 m a.s.l. and a narrower elevation range of 90 m (Table 1). This stratified design captures the major surface conditions in WDL and facilitates cross-surface comparisons of GST across the dominant land-cover types.

**Table 1:** The summary of the 24 ground surface temperature (GST) monitoring sites in the Wudaoliang (WDL) region, central Qinghai–Tibet Plateau (QTP).

Land-cover type	Site	Lon. (°E)	Lat. (°N)	Elev. (m)	Depth	Logger serial number
Alpine desert	AD-1	93.23	35.31	4568	3–5 cm	20672070
	AD-2	93.27	35.31	4543	3–5 cm	20672079
	AD-3	93.27	35.29	4560	3–5 cm	20542329
	AD-4	93.19	35.26	4595	3–5 cm	20672081
	AD-5	93.18	35.26	4611	3–5 cm	20672086
	AD-6	93.12	35.26	4623	3–5 cm	20633677
	AD-7	93.09	35.23	4662	3–5 cm	20633674
	AD-8	93.01	35.08	4692	3–5 cm	20672091
	AD-9	93.00	35.05	4665	3–5 cm	20672099
Alpine steppe	AS-1	93.03	35.32	4705	3–5 cm	20672112
	AS-2	93.09	35.32	4629	3–5 cm	20672073
	AS-3	93.11	35.32	4611	3–5 cm	20672087
	AS-4	93.15	35.32	4606	3–5 cm	20672075
	AS-5	93.19	35.32	4589	3–5 cm	20672078
	AS-6	93.23	35.28	4586	3–5 cm	20672088
	AS-7	93.08	35.22	4619	3–5 cm	20633688
	AS-8	93.09	35.21	4621	3–5 cm	20672085
	AS-9	93.06	35.20	4645	3–5 cm	20672090
Alpine meadow	AM-1	93.02	35.17	4719	3–5 cm	20672089
	AM-2	93.14	35.18	4651	3–5 cm	20672077
	AM-3	93.19	35.16	4667	3–5 cm	20672100
	AM-4	93.22	35.15	4702	3–5 cm	20672074
	AM-5	93.25	35.14	4741	3–5 cm	20633682
	AM-6	93.29	35.14	4681	3–5 cm	20672072

### Instrumentation and installation

GST was measured using HOBO MX2203 TidbiT MX Temperature 400-ft Data Logger (Onset Computer Corporation, USA), equipped with a built-in temperature sensor and programmed to record at 30-minute intervals. The manufacturer-stated specifications for the temperature sensor are a resolution of 0.01 °C, an accuracy of ±0.2 °C over 0–70 °C and ±0.25 °C over −20–0 °C, and a drift of <0.1 °C yr^−1^. All loggers were factory calibrated and were of the same model. Before deployment, the loggers were co-located to screen for obvious inter-logger differences; no anomalous logger was identified, and therefore no additional inter-logger offset correction was applied. Following common permafrost-monitoring usage, GST here denotes ground temperature measured in the uppermost centimeters of the ground^2,7,19^. Sensors were installed at depths of 3–5 cm in hand-augered soil pits, backfilled to maintain stable soil–sensor thermal contact, and covered with the original surface material to avoid radiative heating^39,40^ (Fig. 2d). The logger body remained fully buried under undisturbed soil throughout operation. Each site was georeferenced using a handheld GPS (horizontal accuracy ~3 m; WGS84). The MX2203 loggers are waterproof (IP68) and were deployed without external power, enabling multi-year autonomous operation. Routine status checks and data downloads were performed using the HOBO mobile application via Bluetooth Low Energy (BLE) during maintenance campaigns.

### Data acquisition and preprocessing

Data were downloaded during annual maintenance campaigns (September–October) in 2020, 2021, and 2023. During each visit, sensor position and burial condition were checked, sensors were reburied when necessary, and site conditions were recorded to support subsequent data screening. Export files from multiple downloads were concatenated for each site. Timestamps were standardized to Beijing Time (BJT; UTC + 8; no daylight saving), and duplicate records generated during repeated downloads were removed. All time series were aligned to a common 30-minute timestamp grid. Missing observations and excluded segments were retained as missing values, indicated by blank cells, with no interpolation or gap filling.

### Quality control and frost-jacking exclusion

All GST time series were processed using a harmonized workflow. First, a physical-range screen was applied to flag values outside the plausible interval for near-surface GST in this region, −40 to 50 °C, as invalid. Second, step-change and running-mean shift checks were used to identify sensor drift, abrupt offsets, and dropouts. Specifically, single-step changes >15 °C between consecutive half-hourly samples and persistent level shifts >3 °C in the 24-h running mean were flagged, as such changes are unlikely to reflect physical GST variability alone.

Frost-jacking was the primary field disturbance in WDL, occasionally causing sensor exposure or loss. Potential frost-jacking episodes were first noted during field inspections and then confirmed by manual inspection of the GST time series, supported by diagnostics of the daily GST range, defined as the daily maximum minus minimum. After frost-jacking, the daily range typically increased abruptly in a step-like pattern and remained systematically higher than before, with frequent days exceeding 40 °C. For affected sites, the disturbance-affected period was excluded from the identified onset time onward, or for the documented disturbance window, while the preceding undisturbed records were retained.

To ensure traceability and reproducibility, all excluded periods, including delayed deployment, frost-jacking disturbance, redeployment gaps, and instrument loss, are documented in a machine-readable event log, *qc_event_log.csv*, which records the event type and start and end timestamps of excluded segments, together with brief supporting notes. Users can reproduce the exclusion mask and all analyses reported in this Data Descriptor using the released quality-controlled time series, the event log, and the published scripts.

## Data Records

The WDL GST dataset and associated files are publicly available at the National Tibetan Plateau/Third Pole Environment Data Center^41^. The archive contains four files:

1. *site_info.csv*, a site-level metadata table for all 24 monitoring sites;
2. *gst_half_hourly_data.csv*, the quality-controlled half-hourly GST time series in wide-table format;
3. *qc_event_log.csv*, a machine-readable log of excluded periods and their causes; and
4. *README.txt*, which provides the dataset background, file structure, and variable definitions.

### Site metadata (*site_info.csv*)

Each row corresponds to one monitoring site. The column *site_id* uniquely identifies the site and encodes its dominant land-cover type using a prefix (AD: alpine desert; AS: alpine steppe; AM: alpine meadow). Geographic coordinates are provided as *longitude* and *latitude* in decimal degrees, together with *elevation* in m a.s.l. The columns *start_date* and *end_date* indicate, for each site, the first and last dates of valid observations retained in the released quality-controlled dataset.

### Half-hourly GST time series (*gst_half_hourly_data.csv*)

The file is organized as a wide table in which the first column, *datetime_bjt*, provides timestamps in BJT (UTC + 8) at regular 30-minute intervals. Each subsequent column corresponds to a site, identified by *site_id*, and stores GST in degrees Celsius. Missing values are represented by blank cells, which occur outside the effective observation period of a site (e.g., before deployment or after termination) and for segments removed during quality control. No temporal interpolation or gap filling is applied.

### Traceability of data exclusions (*qc_event_log.csv*)

To make data exclusions transparent and traceable, a machine-readable event log is provided that records, for each site, the event type (e.g., delayed deployment, frost-jacking disturbance, redeployment gaps, or logger loss); the start and end timestamps of the excluded interval in BJT (UTC + 8); and brief evidence or notes describing the basis for exclusion. This log provides an explicit link between excluded periods and the missing-value intervals in *gst_half_hourly_data.csv*, allowing users to interpret data gaps and apply consistent data screening when analysing the quality-controlled time series.

## Technical Validation

### Data completeness and quality-control outcomes

**Fig. 3:**
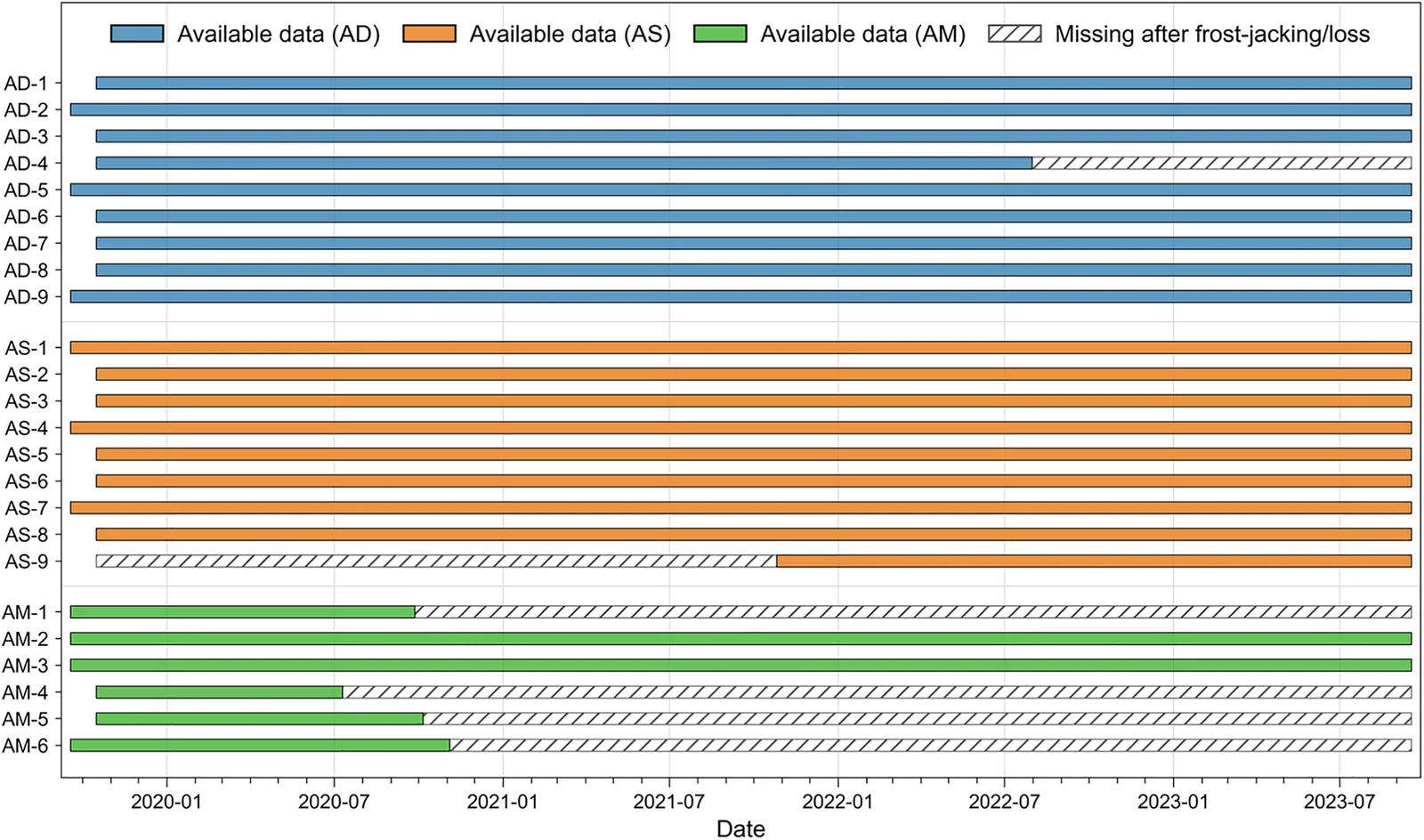
The data availability of the quality-controlled ground surface temperature (GST) time series across the 24 monitoring sites. Colored bars indicate periods with available half-hourly GST observations for each site (AD, alpine desert; AS, alpine steppe; AM, alpine meadow). Hatched segments denote periods missing after frost-jacking disturbance and/or logger loss, including excluded intervals identified during quality control. Missing periods are retained as blank cells in the released dataset, with no gap filling.

After the harmonized quality-control workflow, the released dataset contains 1,399,008 valid half-hourly GST records, corresponding to 83.1% of all possible site–timestamp combinations across the 24 sites over the period from 18 September 2019 to 17 September 2023 (1,399,008 of 1,683,072). Since the establishment of the full network in October 2019, 18 sites provided continuous records through September 2023, whereas six sites (AD-4, AS-9, AM-1, and AM-4–AM-6) have shorter records due to documented field disturbance and/or logger loss, most notably frost-jacking (Fig. 3). Data gaps and excluded segments are retained as missing values in the released time series, and no interpolation or gap filling is applied. The quality-control workflow was designed to remove implausible values and disturbance-affected segments while preserving valid half-hourly measurements.

### Physical plausibility of half-hourly records

**Fig. 4:**
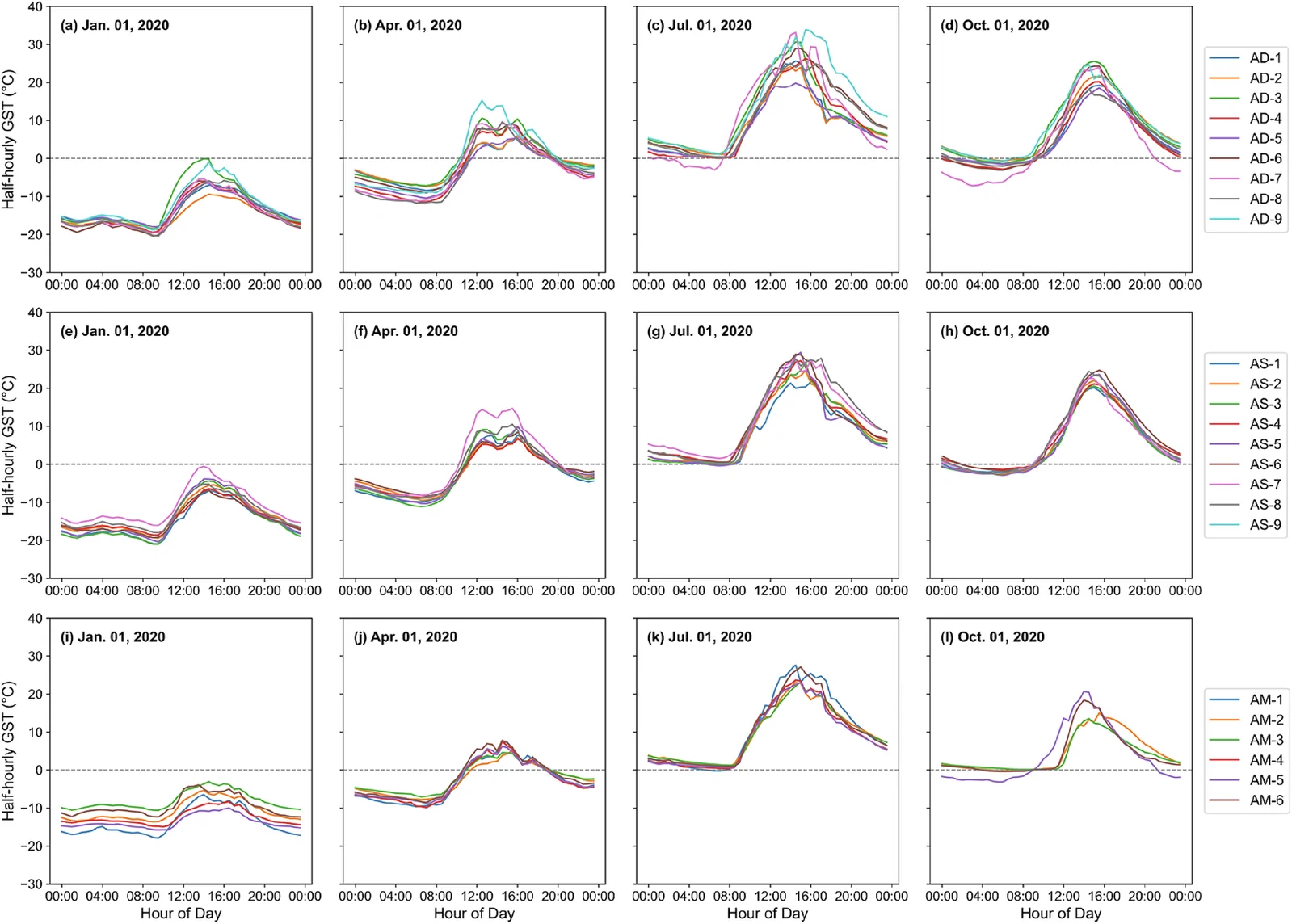
The diurnal ground surface temperature (GST) cycles on four representative dates in 2020. Panels (**a**–**d**), (**e**–**h**), and (**i**–**l**) show alpine desert (AD), alpine steppe (AS), and alpine meadow (AM) sites, respectively. Curves show half-hourly GST in Beijing Time (BJT; UTC + 8) for 1 January, 1 April, 1 July, and 1 October 2020, illustrating typical winter, spring, summer and autumn diurnal behaviour.

To verify the physical plausibility of the quality-controlled half-hourly GST records, representative diurnal GST profiles were inspected on four dates in 2020: 1 January, 1 April, 1 July, and 1 October (Fig. 4). These dates were selected to provide visual checks under typical winter, spring, summer, and autumn conditions on the central QTP. Across sites and land-cover types, the diurnal cycles are smooth and predominantly unimodal, with no isolated spikes or abrupt nonphysical discontinuities. Seasonal progression is coherent: winter curves remain below 0 °C, whereas April and October profiles often approach the freezing point at some sites, consistent with freeze–thaw transition periods. Differences among land-cover types remain within expected surface-dependent variability and do not indicate systematic processing artefacts.

### Internal consistency of daily mean GST

**Fig. 5:**
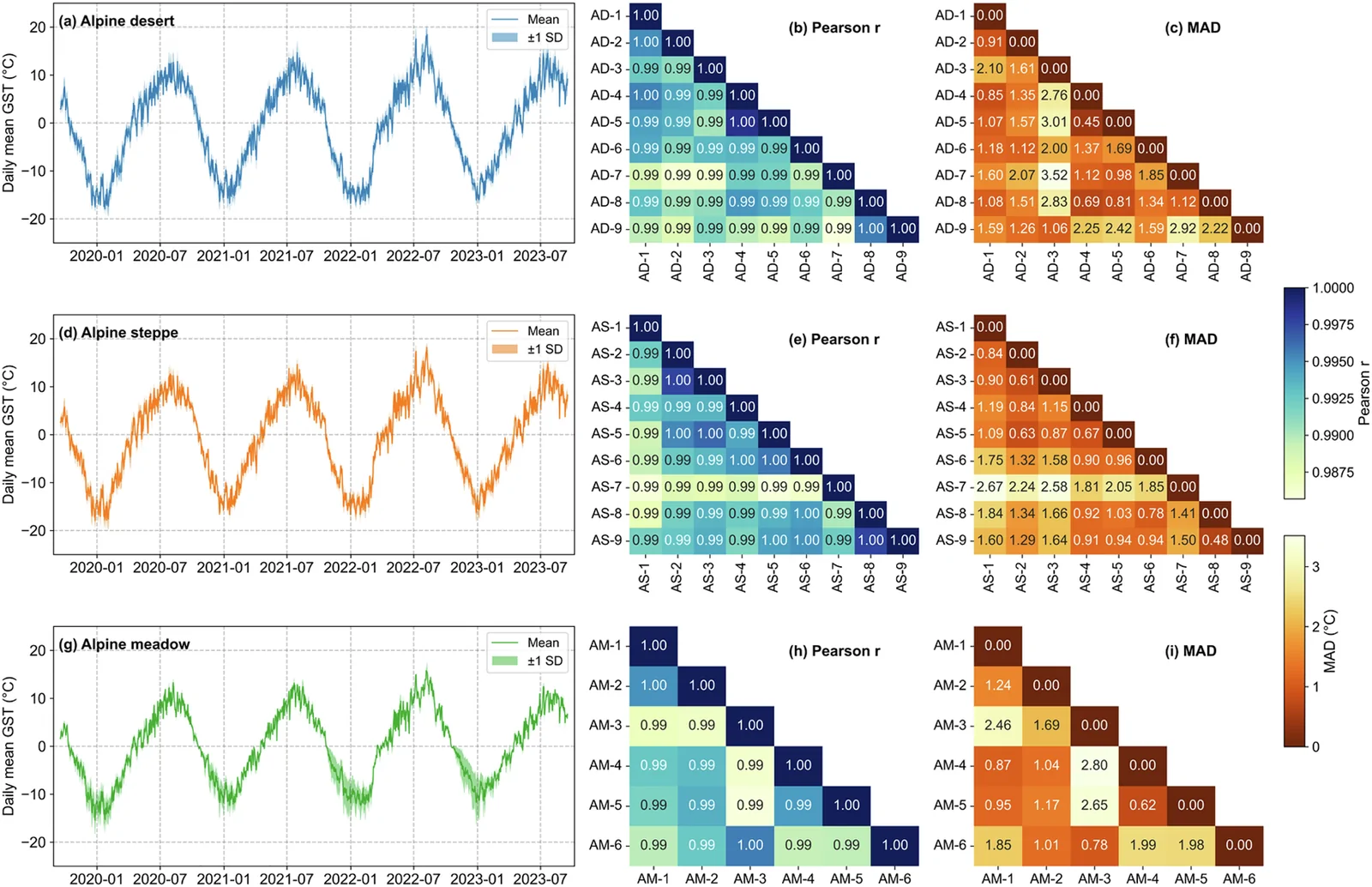
The internal consistency of daily mean ground surface temperature (GST) within each land-cover type. The daily mean GST for (**a**) alpine desert (AD), (**d**) alpine steppe (AS), and (**g**) alpine meadow (AM) sites during 2019–2023. Lines show the across-site mean, and shading indicates ± 1 standard deviation. Pairwise agreement is summarized by Pearson’s correlation coefficient (r) in (**b,e,h**) and mean absolute difference (MAD) in (**c,f,i**), calculated over overlapping periods with data at both sites.

To assess internal consistency of the released dataset, daily mean GST derived from the quality-controlled half-hourly series was evaluated by examining continuity and agreement among sites within each land-cover type (Fig. 5). Daily mean GST evolves smoothly over 2019–2023 at all sites, with no step-like jumps, gradual drift, or isolated spikes after quality control. Between-site agreement was quantified using the pairwise Pearson’s correlation coefficient (r) and mean absolute difference (MAD) computed over overlapping periods. Pearson’s r exceeds 0.99 for all site pairs within each land-cover type. The MAD values are 1.6 ± 0.7 °C for AD, 1.3 ± 0.5 °C for AS, and 1.5 ± 0.7 °C for AM, with maxima of 3.5, 2.7, and 2.8 °C, respectively. These statistics indicate strong temporal coherence among nearby sites subject to common synoptic forcing, while retaining realistic site-to-site differences.

### Internal consistency of daily GST range

**Fig. 6:**
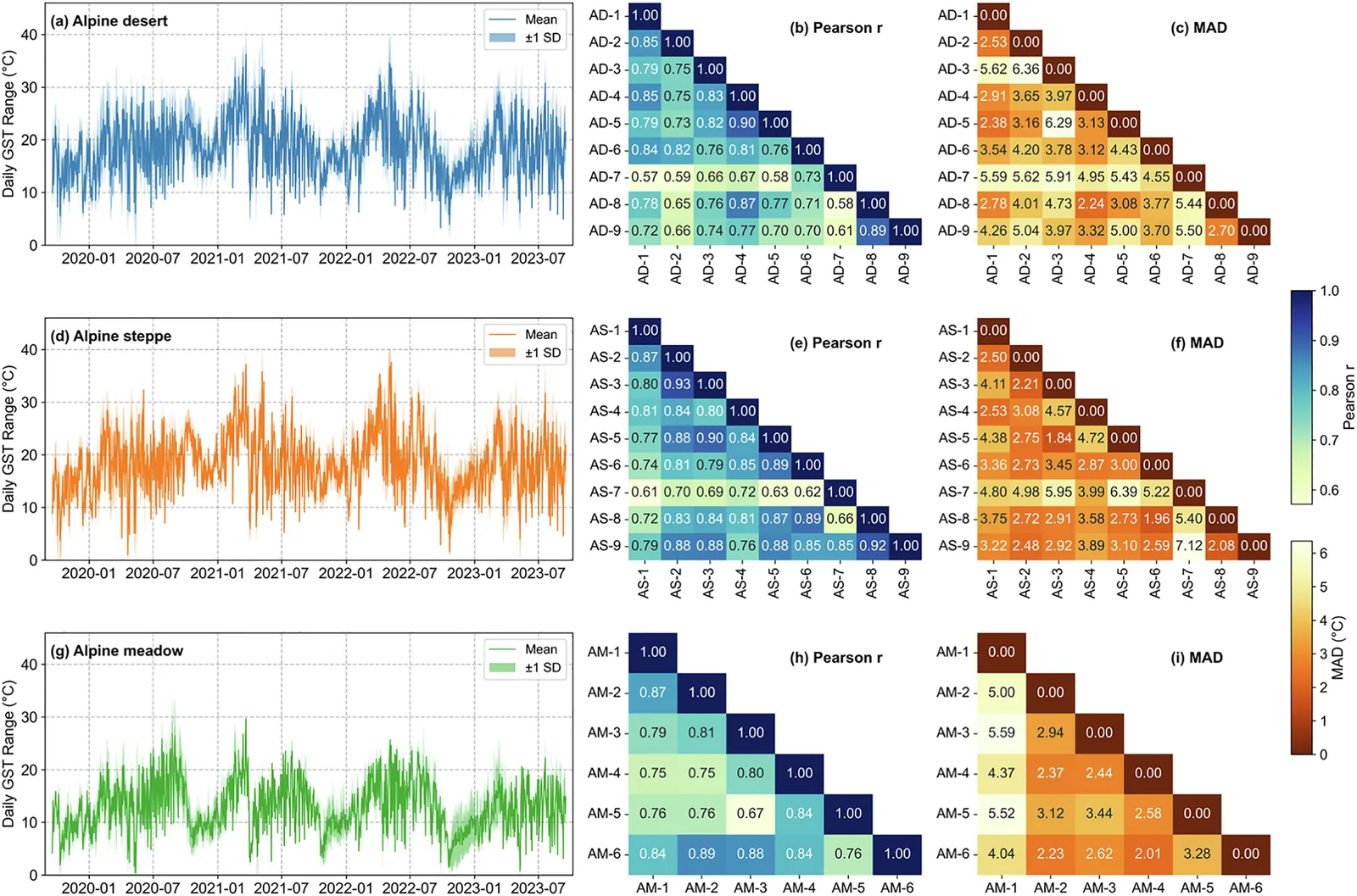
The internal consistency of daily ground surface temperature range (DGSTR) within each land-cover type. The daily DGSTR for (**a**) alpine desert (AD), (**d**) alpine steppe (AS), and (**g**) alpine meadow (AM) sites during 2019–2023. Lines show the across-site mean, and shading indicates ± 1 standard deviation. Pairwise agreement is summarized by Pearson’s correlation coefficient (r) in (**b,e,h**) and mean absolute difference (MAD) in (**c,f,i**), calculated over overlapping periods with data at both sites.

As a complementary check sensitive to high-frequency variability and potential disturbance effects, the daily GST range (DGSTR; daily maximum minus daily minimum) was analyzed from the half-hourly series (Fig. 6). DGSTR exhibits coherent seasonal evolution across sites within each land-cover type. Pairwise agreement is lower than that for daily mean GST, as expected for a metric influenced by local surface conditions and microscale heterogeneity, but remains broadly consistent across the network. Land-cover-type mean pairwise correlations range from 0.75 to 0.80, with corresponding mean pairwise MAD values of about 3.4–4.2 °C. Large but physically plausible DGSTR values indicate that the quality-control procedure did not artificially smooth valid high-frequency variability.

### Limitations

The following limitations should be considered when interpreting or reusing the released GST records. GST in this release was measured at 3–5 cm depth and should not be treated as a direct *in situ* equivalent of satellite “skin” LST. This measurement depth should be considered when comparing the dataset with remote-sensing products or model variables defined at different depths. Data gaps primarily reflect field disturbance rather than processing artefacts: six sites were affected by frost-jacking and/or logger loss, resulting in shorter records and gaps that are retained as missing values, with no interpolation. The relatively higher data loss at AM sites likely reflects the susceptibility of shallowly buried loggers to freeze–thaw disturbance in wetter meadow soils. Frost heave and possible ice-lens development may reduce soil–sensor contact, move loggers upward, or expose them at the ground surface. Future deployments in comparable meadow environments should consider paired or redundant shallow loggers at selected sites, careful burial and backfilling, robust site marking and installation records, post-freeze/thaw inspections where feasible, and clear documentation of disturbance-affected periods. Spatial representativeness is constrained to the WDL region and its dominant land-cover types, which are likely more representative of high-elevation, relatively arid parts of the Plateau than wetter eastern environments. Finally, fine-scale heterogeneity, including microtopography, vegetation structure, soil texture, and soil moisture, can produce site-to-site differences when comparing point measurements with gridded products.

## Usage Notes

### Recommended aggregation and seasonal summaries

The released GST series are provided at 30-minute intervals on a regular timestamp grid. In this release, any day with available data contains a complete set of 48 half-hourly observations from 00:00 to 23:30 BJT, so daily diagnostics (e.g., daily mean and daily range) can be computed directly from the half-hourly series, with missing periods retained as missing intervals. For freeze–thaw and degree-day applications, hydrological-year summaries (e.g., freezing and thawing degree days) are often more informative than calendar-year summaries. Notably, 18 sites provided continuous coverage of three thaw seasons and four freezing seasons during 2019–2023. The observation period also captured the 2022 extreme summer heatwave in the central QTP^42^, enabling the quantification of thermal extremes and associated changes in near-surface ground thermal conditions.

### Examples of satellite product applications

#### Pairing with MODIS LST

**Fig. 7:**
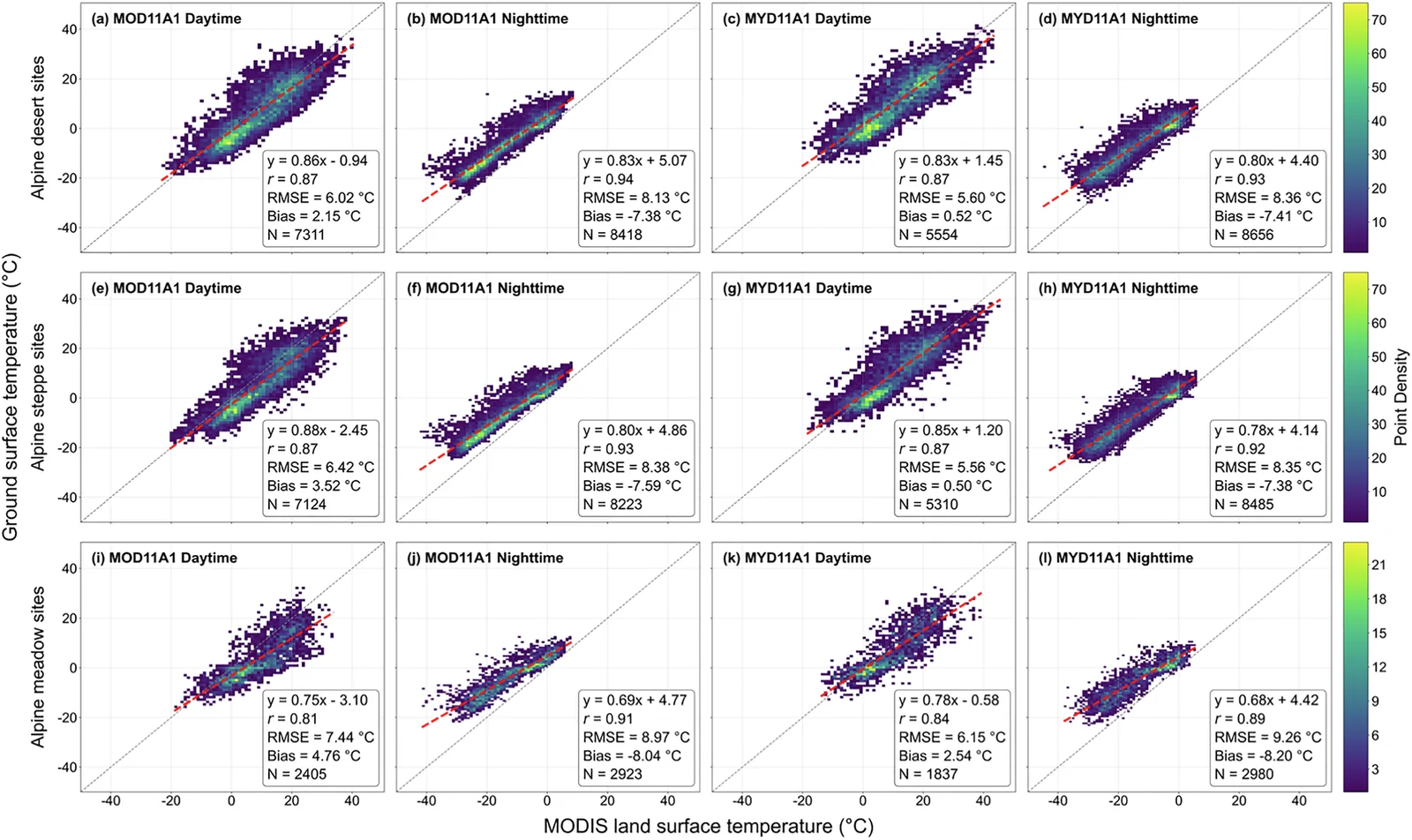
The density scatterplots comparing MODIS land surface temperature (LST) and *in situ* ground surface temperature (GST; measured at 3–5 cm depth) for 2019–2023. Panels show results stratified by land-cover type (**a**–**d**, alpine desert; **e**–**h**, alpine steppe; **i**–**l**, alpine meadow), platform (Terra MOD11A1; Aqua MYD11A1; Collection 6.1), and overpass time (daytime versus nighttime). Matched pairs were constructed by selecting valid-quality MODIS retrievals and pairing each overpass with the nearest half-hourly GST observation within ±15 min. Point density is indicated by color.

Users can pair the half-hourly GST series with daily 1-km MODIS LST products from Terra (MOD11A1) and Aqua (MYD11A1), Collection 6.1. MODIS pixel values should be extracted at each site location, or as a small-neighbourhood average if geolocation uncertainty is a concern, converted to °C, and retained only for valid-quality retrievals based on MODIS QA flags. Each satellite overpass can be matched to the temporally nearest *in situ* GST observation within a narrow time window (e.g., ±15 min) using the nominal MODIS observation time referenced to local standard time. Because retrieval characteristics differ by platform and overpass time, pairing and any subsequent calibration should be stratified by daytime versus nighttime and by Terra versus Aqua, and may also be stratified by land-cover type, including AD, AS, and AM. Matching rules, including the time window, QA screening, spatial sampling scheme, and stratification, as well as the resulting sample sizes, should be reported to ensure reproducibility. Figure 7 illustrates an example GST–MODIS pairing.

#### Freeze–thaw product comparison

**Fig. 8:**
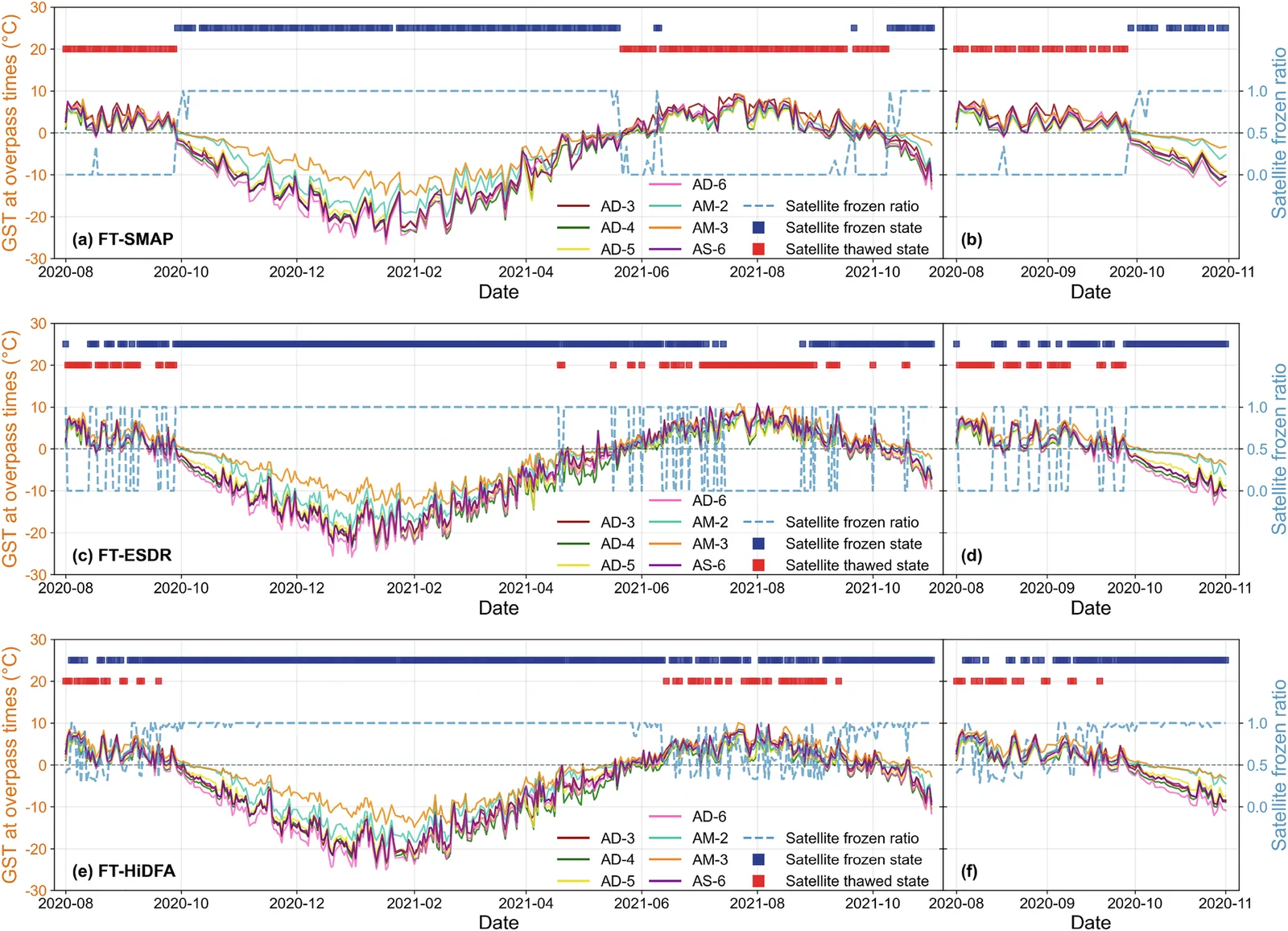
An example comparison of ground surface temperature (GST) with satellite freeze–thaw (FT) products at the grid-cell scale. Comparisons are shown for three FT products (FT-SMAP^43^, FT-ESDR^44,45^, and FT-HiDFA^46,47^) within a 25-km EASE-Grid cell (row 124, column 1054) containing seven GST sites. Panels (**a,c,e**) show GST time series as colored lines, together with the satellite frozen ratio shown by the dashed blue line on the right axis and the corresponding binary FT state shown as bars at the top. Panels (**b,d,f**) provide zoomed views of the autumn transition period (August–October 2020), illustrating sensitivity to overpass timing and frequent near-0 °C crossings.

Figure 8 illustrates an example comparison between GST–satellite freeze–thaw (FT) products using two widely used global FT datasets (FT-SMAP^43^, FT-ESDR^44,45^) and a recently released higher-resolution product (FT-HiDFA^46,47^), evaluated within a common 25-km EASE-Grid cell containing seven GST sites. For reproducibility, each satellite overpass should be paired with the temporally nearest *in situ* GST measurement and classified using a simple threshold (frozen if GST ≤ 0 °C and thawed otherwise). Where products provide sub-grid information, users may also summarize spatial variability using a frozen ratio, defined as the fraction of frozen sub-grid pixels, alongside the binary FT state. Particular care is needed during spring and autumn transitions, when GST often fluctuates around 0 °C and threshold crossings can be frequent. Results can be sensitive to the pairing time window, overpass timing (ascending/descending), and whether instantaneous GST or aggregated metrics are used. Reporting site-level outcomes and stratifying by land-cover type can help separate product behavior from unresolved sub-grid heterogeneity.

### Long-term GST reconstruction

The GST dataset provides a local benchmark for reconstructing longer-term GST series from multi-year air-temperature records, thereby extending GST forcing beyond the *in situ* observation period for applications such as permafrost modelling (e.g., Sun *et al*.^25^). This represents a potential downstream use of the dataset; specific reconstruction methods and model configurations are application-dependent and are therefore not detailed in this Data Descriptor.

## Data Availability

The quality-controlled half-hourly GST dataset, site metadata, quality-control event log, and README file are publicly available at the National Tibetan Plateau/Third Pole Environment Data Center: https://doi.org/10.11888/Cryos.tpdc.303271.

## Code availability

No new algorithms were developed. Python 3 scripts were used for quality control, derivation of the quality-controlled half-hourly and daily GST products, and reproduction of all analyses and figures reported in this Data Descriptor. The code, with environment specifications and a run guide, is archived at the National Tibetan Plateau/Third Pole Environment Data Center https://doi.org/10.11888/Cryos.tpdc.303271^41^.
